# Self-administered questionnaire versus interview as a screening method for intimate partner violence in the prenatal setting in Japan: A randomised controlled trial

**DOI:** 10.1186/1471-2393-10-84

**Published:** 2010-12-24

**Authors:** Yaeko Kataoka, Yukari Yaju, Hiromi Eto, Shigeko Horiuchi

**Affiliations:** 1 St Luke's College of Nursing, 10-1 Akashi-cho, Chuo-ku, Tokyo 104-0044, Japan Associate Professor in nursing and midwifery; 2 Department of Medicine, Pharmacoepidemiology, University of Tokyo, 7-3-1 Hongo, Bunkyo-ku, Tokyo 113-0033, Japan

## Abstract

**Background:**

Intimate partner violence (IPV) is a serious social issue in Japan. In order to start effective interventions for abused women, the appropriate method of screening for IPV in healthcare settings needs clarifying. The objective of this study was to compare the effectiveness of a face-to-face interview with a self-administered questionnaire. We used the Violence Against Women Screen (VAWS), a Japanese screening instrument for intimate partner violence (IPV), for identifying pregnant women who have experienced abuse.

**Methods:**

We conducted a randomised controlled trial to screen participants at three points in time in a prenatal clinic in Tokyo, Japan. There were 328 consenting women between 14 and 25 weeks of pregnancy who were consecutively selected and randomly assigned to either the interview or self-administered questionnaire group. Both groups completed the same screening instrument three times during their pregnancy. The primary outcome was the total number of women identified by each screening method and the secondary outcome was the effect of the screening as measured by the women's comfort level and their expressed need to consult with the nurse.

**Results:**

For all three screenings, the identification rate in the interview group was significantly lower than that for the self-administered questionnaire group (relative risk 0.66, 95% CI 0.46 to 0.97), even after controlling for smoking (adjusted odds ratio 0.59, 95% CI 0.35 to 0.98). The two groups did not differ for secondary outcomes.

**Conclusions:**

The self-administered questionnaire identified more IPV than the face-to-face interview when screening pregnant women in a Japanese prenatal clinic.

**Trial Registration:**

UMIN-CTRC000000353

## Background

Intimate partner violence (IPV) is a serious social issue in Japan. A national survey conducted in 2008 revealed that approximately 25% of adult women, an estimated eight million women, reported being subjected to physical violence by a male partner, over their lifetime: approximately 16% reported sexual violence, and approximately 17% reported psychological abuse [1]. This survey revealed that the 5-year prevalence of IPV was 14% among women. Furthermore the number of women experiencing physical, sexual or psychological violence from a partner has increased gradually, approximately 19% to 33%, over the ten-year period of 1998-2008 [1,2].

The law for the prevention of spousal violence and the protection of victims, enacted in Japan in 2001, called for the identification of abused women and for providing adequate resources such as support centres or hotlines. The language used in the law indicates that health care providers may report IPV to the police and support centres, taking into consideration the intention of the victim, however reporting is not mandated. Providing women with access to counselling centres is a clear intent of the law. Despite the law, most health care providers are not adequately trained to carry out this role [3], nor is abuse screening, or even inquiry, routine in the healthcare settings, therefore safety planning and referral to community resources are not provided. It is also likely that many health professionals do not recognize most incidents of IPV and therefore do not contribute to the woman's health recovery.

According to a recent survey in Japan, the prevalence of IPV during pregnancy was around 5% [4]. IPV affects women and infant's health and pregnancy outcomes: gynecological symptoms [5], sexually transmitted diseases, abortion, and complications of pregnancy: diabetes, hypertension, infection, anemia [6-8] and low weight infants [9]. Pregnancy is one of the few opportunities where healthy women come into contact with their own health care providers. Therefore, IPV screening and assessment in prenatal settings should be standard care for all pregnant women. Although there is insufficient evidence to support the effectiveness of interventions after universal screening [10,11], screening, carefully orchestrated early intervention attempts during pregnancy for IPV are still imperative because of the high risk of substantial damage to the health of women and children. In order to start effective interventions for abused women in Japan, the appropriate timing and method of IPV screening in healthcare settings needs to be better understood.

Even though researchers developed valid and reliable screening instruments [12], their effective implementation is still controversial. Researchers have conducted some randomised controlled trials (RCT) to compare screening methods. MacMillan et al. examined optimal screening methods (computer, written, face-to-face) for IPV in emergency departments, family practice and women's health clinics. They concluded that no statistically significant differences were found for screening methods, however, women preferred the self-administered method [13]. Another RCT in family practice also identified no statistically significant difference between interviews and self-administered questionnaires [14]. Of the two RCTs conducted in emergency departments, one demonstrated that computerized screening increased the identification rate of abused women compared to a questionnaire [15]; the other found that an audiotape questionnaire detected more abused women than a questionnaire [16].

All RCTs were conducted in North America; no studies were undertaken in Asian countries or in prenatal settings. The issue of cultural sensitivity remains unexplored. It is not known whether approaches used in North American are relevant in Asian countries. While the names of the cultural values may be similar, such as respect, trust and caring, the order and meaning of the values and the way they are enacted may differ, thus requiring different approaches in screening for domestic violence. Therefore we conducted a randomised controlled trial in a prenatal clinic in Japan to compare the rate of IPV disclosure based on face-to-face interviews and a self-administered questionnaire and to identify responses to screening experiences.

## Methods

This was a parallel group, randomized controlled treatment study. The study took place from February through November 2003 at the prenatal clinic of a general hospital, in a typical urban area of Tokyo, which had approximately 1500 deliveries each year.

### Participants

Eligible pregnant women were consecutively selected between February 22 and May 30, 2003. At the time of recruitment most were at 14-15 weeks of pregnancy, although a few were close to 25 weeks, which was the cut-off point for study eligibility. Research assistants, who had master's degrees in nursing, or the researcher (YK) invited eligible women to privately discuss their participation in the study. They were informed in detail (following the Declaration of Helsinki) regarding their right to confidentiality, to withdraw from the study at any time without jeopardizing their care and protection of anonymity if results were published or presented. The women were asked if they were Japanese speaking, had any serious mental diseases and if their partner would agree to leave the room during the interview or while completing the questionnaire. Children under two years old were allowed to stay with their mother, but older children, who might report the discussion about IPV to the male partner or family members [17], were not allowed to stay and instead were provided with toys and a separate place to play. All participants gave written informed consent.

### Randomization

After informed consent, the participants were randomised, by means of numbered sealed envelopes, to either the interview or the questionnaire group. We used a random number table in blocks of four to ensure that approximately equal numbers of women were allocated to each group. Because of the nature of the screening methods participants could not be blinded to the group assignment. The same researcher performed the allocation procedure and data analysis.

### Instruments

Two methods of implementing the screening tool, Violence Against Women Screen (VAWS), were compared in this trial: self-administered and face-to-face interview. VAWS is a 7-item Japanese screening instrument. Items include information about: couple's relationship, perceived behavior of the partner (i.e. difficultly settling arguments by talking it through; becoming frightened, being yelled at, partner hitting the wall or throwing objects; being forced into sex; being pushed or slapped; being hit or kicked in that order). Item responses are on a 3-point Likert scale: *often *(2), *sometimes *(1) or *never *(0). The scores range from 0 to 14 and a score of 2 or above indicates IPV. The sensitivity of VAWS is 86.7% and specificity is 80.2%, based on the Japanese version of the index of spouse abuse [18].

Secondary outcomes were comfort level and the need to consult with the nurse after screening, which were used as indicators of possible adverse effects of IPV screening. The comfort level question referred to their sense of ease or discomfort. We asked women who had used either method: self-administered questionnaire or interview: "How did you feel being questioned about your relationship with your partner". They indicated their comfort level using a 4-point Likert rating: 1 (*not at all comfortable*) to 4 (*very comfortable*). In order to measure their desire for consultation about their relationship, we asked women: "Would you like to consult with a nurse when you have a problem with your husband?" The response options were: *yes*, *no *or *cannot decide*.

Prior to administrating the first screening, all participants responded to a written questionnaire covering demographic information such as age, education, employment, marital status, alcohol and smoking habits before pregnancy, and lifetime experience of physical violence from a former partner.

### Screening and consultation protocol

All participants received three screenings: the first screening was upon entry to the study at 14-25 weeks of pregnancy; the second screening was at 20-30 weeks and the third screening was at 35 weeks or more. In the first screening, women reported about abuse for the "past 12 months", but at screenings two and three, they reported about abuse: "since the last time you were interviewed" [19]. Three assessments were made because disclosure about abuse could occur at any time during pregnancy especially after establishing a trusting relationship with a nurse.

The interviewers were three female nurses working at the hospital outpatient department. The researcher provided three training sessions in order to assure interviews proceeded in accordance with the study protocol and with an awareness of IPV as an issue for women. The first training session was a 2-hour lecture about the prevalence, dynamics and the physical and psychological impact of IPV, and appropriate care for abused women. A survivor of IPV who told her story of recovery conducted the second session. Theories (i.e. cycle of violence and learned helplessness) were discussed to explain why she did not escape from her partner. In the third session, the researcher trained the nurses on the use of the protocol for screening and intervention for abused women in the prenatal setting.

Interviews were conducted at the prenatal clinic in a private room that was partitioned into three cubicles or in a private area. The nurse sought to establish a partnership with the woman, based on the women centered care model [20] of respect and empowerment. Half of the women were interviewed by the same nurse at all three screenings. The nurse read the VAWS questions to the woman, while the woman also read the questionnaire.

If the woman screened positive and wanted the nurse to report the abuse to a support centre or to the police, the nurse would make the report. In addition, the nurse discussed with the woman how to maintain her safety and attempted to aid the woman in decision-making. The nurse also gave the woman a community resource card with information about a crisis hotline and local shelters. Women in the questionnaire group completed the VAWS in the official prenatal clinic interview room where the same type of resource cards were available. The resource cards were also available in the waiting area. The abuse consultants' contact information was listed in the questionnaire for easy access. Thus, although all the women had access to IPV resources the women in the interview group may have received additional assistance compared to the women in the questionnaire group.

### Data Collection

The primary outcome was the disclosure rate of abuse by women who were identified by the VAWS tool during the three screening sessions compared to those in the interview group. We counted single disclosures only once, at the first or second or third screenings. We also included the pattern of multiple disclosures at screenings: first and second; first and third; second and third and first, second and third. This provided us with unduplicated accounts of abuse disclosures.

### Sample Size and Statistical Analysis

From the survey, we estimated the baseline proportion of physical violence cases during pregnancy to be 3% [4], so we aimed to recruit 140 women per group in order to detect a difference between groups at a 7% effect size, based on previous research [19,21] with 80% power. Extra women were recruited to account for attrition, which was expected to be 10-20%.

All analyses were carried out using SPSS for Windows 11.5 J (Chicago, IL, USA). Categorical differences between the two groups were tested with the χ^2 ^test or Fisher's exact test. Multiple logistic regression analysis was performed to adjust for potential baseline differences in the characteristics between the groups. For each group we calculated the IPV positive screen rate using a 95% confidential interval (CI). All statistical tests were performed with a two-sided 5% level of significance. All analyses were done using an intention-to-treat basis, which included all randomized participants. The Ethics Committee of St.Luke's College of Nursing approved the protocol.

## Results

### Sample and Demographic Characteristics, and Retention Rates

**Figure 1:**
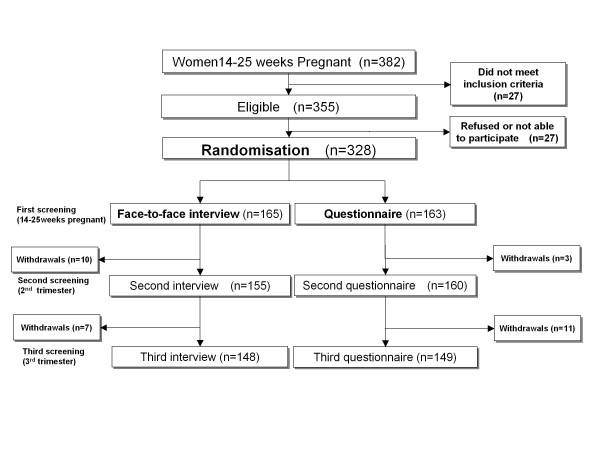
**Flowchart of the random control trial process**.

During the study period, 382 pregnant women made an appointment at the hospital between 14 and 25 weeks of their pregnancy (Figure 1). Of these, 355 (92.9%) met all eligibility criteria; eight were lost to follow-up and 19 refused to participate, the remaining 328 eligible women were randomly assigned to either the interview or questionnaire group. With the exception of smoking, there were no significant differences between the groups at baseline (Table 1) and the primary outcome was adjusted to account for this. In the interview group, 148 (89.7%) participants completed all three screenings and 17 withdrew because of: referral to university hospitals (n = 3); moving out of the area (n = 9); preterm delivery (n = 1) or refusal to continue the screening interview (n = 2). In the questionnaire group, 149 (91.4%) participants completed all three screenings and 14 withdrew because of moving out of the area (n = 13) or preterm delivery (n = 1). Retention rates did not differ between the two groups.

**Table 1:** Demographic characteristics of the women at first screening during the first trimester of pregnancy (N = 328)

	Face to face interview group (n = 165)	Questionnaire group (n = 163)
Age (years)		
20-29	47 (29.0)	53 (32.7)
30-39	111 (68.5)	106 (65.4)
≥40	7 (2.5)	3 (1.9)
Parity		
Primipara	102 (62.6)	105 (64.4)
Multipara	61 (37.4)	58 (35.6)
Education		
Junior high/High school graduate	20 (12.3)	24 (14.8)
Junior college graduate	74 (45.7)	69 (42.6)
College graduate/Postgraduate	68 (42.0)	69 (42.6)
Marital status		
Married	159 (98.1)	157 (97.5)
Single	3 (1.9)	4 (2.5)
Employment		
Full time	53 (32.7)	58 (36.40)
Part time	32 (19.8)	26 (16.1)
Not working	77 (47.5)	77 (46.9)
Alcohol before pregnancy		
Yes	109 (67.3)	115 (71.0)
No	53 (32.7)	47 (29.0)
Smoking before pregnancy		
Yes	42 (25.9)	27 (16.7)
No	120 (74.1)	135 (83.3)
Lifetime experience of physical violence by male partner		
Yes	8 (4.8)	11 (6.8)
No	154 (95.1)	151 (93.2)

Values are number (percentage).

### Primary Outcome

For the interview group the identification rate was 32/165 (19.4%) compared with 48/163 (29.4%) in the questionnaire group, which was much higher (relative risk 0.66, 95% CI 0.46 to 0.97), even when controlled for smoking (adjusted odds ratio 0.59, 95% CI 0.35 to 0.98).

The numbers of abused women disclosing abuse at each screening are shown in Table 2. Of the 32 women in the interview group, 16 (50.0%) disclosed abuse only at the first screening compared to seven (14.6%) from the questionnaire group. There was no discernable disclosure pattern between the two groups or within each group.

**Table 2:** Number of women disclosing abuse at each screening by group

Time of disclosure	Face to face interview group (n = 165)		Questionnare group (n = 163)		Relative risk	(95%CI)	P value*
**Total no. abused women in the 3 screenings**	**32**		**48**		**0.66**	**(0.45-0.97)**	**0.034**

First screening only	16	(50.0)	7	(14.6)			
Second screening only	1	(3.1)	5	(10.4)			
Third screening only	3	(9.4)	1	(2.1)			
First and second screening	2	(6.3)	10	(20.8)			
First and third screening	3	(9.4)	4	(8.3)			
Second and third screening	1	(3.1)	4	(8.3)			
First, second and third screening	6	(18.8)	17	(35.4)			

Values are number (percentage) unless stated otherwise

Numbers are unduplicated

*χ^2 ^test

### Secondary Outcomes

The comfort level during screening for DV did not differ between the two groups: 98 (68.1%) women in the interview group and 88 (60.7%) in the questionnaire group responded that they were "very comfortable" with the screening for DV. The rate of those needing consultation with a nurse after the screening was similar and relatively low in both groups (interview 11.1%; questionnaire 11.7%).

## Discussion

### Implications of the Findings

The results of a RCT conducted to determine which of two screening methods (face-to-face interview or self-administered questionnaire) was more effective, in detecting woman who experienced abuse from their partner, in the prenatal setting in Japan indicated that the questionnaire method identified more abused women than the interview.

Previous studies of obstetric patients demonstrated higher disclosure with written questionnaires compared with face-to-face interviews [21,22], although other RCTs reported no difference among methods [13,14]. The results of our study concur with those of Caterino et al. [21] and Webster et al. [22] who studied pregnant women as did this study. Pregnant women usually come to the prenatal clinics with their partners, so they are more cautious about privacy. This is the main reason that a written questionnaire is optimal for pregnant women especially in Japan, a very homogeneous country where the literacy rate is almost 100%. In addition the VAWS is written in an easy form of Japanese.

Cultural reasons must also be considered as to why the self-administered questionnaire was the better method for pregnant women to identify IPV in this prenatal setting. First, for Japanese, maintaining harmony in a group is a strong value; therefore open communication is discouraged because of the group orientation [23]. There is also a strong value of keeping family matters private and inner feelings hidden [23]. This means it is difficult to disclose private matters verbally to another person, especially concerning problems within a family, even if the other person is a health professional. With regards to IPV, women tend to feel shame and guilt, so disclosing though a face-to-face interview is even more difficult. In contrast, privately filling out the written questionnaire might seem easier and more secure when revealing partner violence. Second, healthcare settings in Japan still lack privacy; for example, waiting rooms are crowded and there are few rooms for private consultation. Even in this study, in which the privacy of the women during the interview was our first concern; we provided privacy but could not always provide a private room. Finally, the face-to-face interview requires on-going training and extra time; it would require a national level initiative to establish that resource within prenatal settings.

We found that the proportion of new, ended and ongoing cases was different between the two groups. About one third of abused women in the questionnaire group reported violence over time. By contrast half of abused women in the interview group reported violence only at the first screening. Previous studies revealed it was difficult to change the situation or relationship between a couple, and to stop the violence in this short period of time [24]; therefore it is possible that some women were reluctant to continue to admit to being abused. Social desirability effect might be the cause of this difference. In addition although most of the women who were interviewed said that they felt "comfortable" with the screening, they might have responded differently if there were more questions about aspects of comfort. Finally, previous studies revealed that the effect of training was not maintained 12 months later [25]. The effect of training was not evaluated in the present study and the care for the women may have changed over the 10-month study period so that the interviewers were less supportive during the interview.

It is important to ensure that pregnant women feel safe answering IPV screening questions. In Japan, we recommend that women screened as positive discuss their situation and feelings after the self-administered questionnaire screening. Identifying abused women enables the IVP trained healthcare provider to intervene, providing both the woman and fetus with a safer environment. The nursing care protocol for abused women that was used in this study needs to be further developed in order to improve prenatal care in Japan.

### Study Limitations

First, the study protocol excluded women who: were non-Japanese speakers; had a serious mental illness; did not agree to leave their partner during the interview, and an additional 27 women who refused to participate. This may have generated a self-selection bias eliminating women who were at high-risk for DV leading to underestimation of its prevalence. Second, measurements of primary and secondary outcomes had psychometric property limitations. The VAWS was the first Japanese IPV screening instrument that was developed with respect to culture and social situation. Although the sensitivity of the VAWS was comparatively high, specificity was low compared with other tools such as the: 'Hurts, Insults, Threatens and Screams at Her' (HITS), Women's Experience with Battering Scale (WAST) and Partner Violence Screen (PVS). We need additional valid and reliable Japanese screening tools to test the screening methods. Measurements of secondary outcomes were also limited by their reliability and validity. The concepts of 'comfort' and 'need' require further development and testing within the context of the Japanese prenatal setting. A single question might be not enough to measure the comfort level and needs, even though Sauro & Dumas found acceptable reliability and validity with one-question Likert tests [26]. We will have to detect if adverse effects occurred in one method of screening more than the other. Third, this study was conducted at an urban prenatal clinic. Further studies are needed in different geographical areas and in different types of healthcare settings, such as emergency departments or mental health care clinics, to establish the generalization of the results. However, these limitations do not detract from the important finding of this study, which is that a self-administered questionnaire resulted in more disclosure of IPV in a Japanese prenatal setting. Finally, although the study was conducted in 2003, the data remains useful and fortunately still quite timely. These findings should encourage researchers in Asian countries to replicate and extend the research.

## Conclusion

In Japan, within a perinatal setting, the self-administered questionnaire compared to the face-to-face interview detected significantly more women who experienced abuse from their partners. The comfort level and the need for consulting with a nurse after the screening did not differ between the two groups and must be interpreted with caution because of the beginning stage of tool development. In conclusion the self-administered questionnaire was an appropriate method for identifying abuse in a Japanese prenatal setting when conducted with privacy safeguards and resource follow-up.

## Competing interests

The authors declare that they have no competing interests.

## Authors' contributions

YK carried out the data collection and drafted the manuscript. HE participated in the sequence alignment. YY participated in the design of the study and performed the statistical analysis. SH conceived of the study, and participated in its design and coordination and helped to draft the manuscript. All authors read and approved the final manuscript.

## Pre-publication history

The pre-publication history for this paper can be accessed here:

http://www.biomedcentral.com/1471-2393/10/84/prepub
